# A nicotinic acetylcholine receptor transmembrane point mutation (G275E) associated with resistance to spinosad in *Frankliniella occidentalis*

**DOI:** 10.1111/jnc.12029

**Published:** 2013-01-13

**Authors:** Alin M Puinean, Stuart J Lansdell, Toby Collins, Pablo Bielza, Neil S Millar

**Affiliations:** * Department of Neuroscience, Physiology & Pharmacology, University College LondonLondon, UK; † Departmento de Producción Vegetal, Universidad Politécnica de CartagenaCartagena, Spain

**Keywords:** *Frankliniella occidentalis*, insecticide resistance, nicotinic acetylcholine receptor, spinosad

## Abstract

High levels of resistance to spinosad, a macrocyclic lactone insecticide, have been reported previously in western flower thrips, *Frankliniella occidentalis*, an economically important insect pest of vegetables, fruit and ornamental crops. We have cloned the nicotinic acetylcholine receptor (nAChR) α6 subunit from *F. occidentalis* (Foα6) and compared the nucleotide sequence of Foα6 from susceptible and spinosad-resistant insect populations (MLFOM and R1S respectively). A single nucleotide change has been identified in Foα6, resulting in the replacement of a glycine (G) residue in susceptible insects with a glutamic acid (E) in resistant insects. The resistance-associated mutation (G275E) is predicted to lie at the top of the third α-helical transmembrane domain of Foα6. Although there is no direct evidence identifying the location of the spinosad binding site, the analogous amino acid in the *C. elegans* glutamate-gated chloride channel lies in close proximity (4.4 Å) to the known binding site of ivermectin, another macrocyclic lactone pesticide. The functional consequences of the resistance-associated mutation have been examined in the human nAChR α7 subunit. Introduction of an analogous (A272E) mutation in α7 abolishes the modulatory effects of spinosad whilst having no significant effect upon activation by acetylcholine, consistent with spinosad having an allosteric mechanism of action.

Spinosad is a macrocyclic lactone, isolated from the microorganism *Saccharopolyspora spinosa* (Sparks *et al*. 1998; Thompson *et al*. 2000). It is a naturally occurring mixture of two components, spinosyn A and spinosyn D (Fig. 1) and was introduced as a commercial insecticide in 1997 (Thompson *et al*. 2000). Spinosad is used extensively in crop protection to control a wide range of insect pests, including lepidoptera and thysanoptera, but it is also used in animal health applications and to control head lice in humans. Insect toxicity is associated with widespread neuronal excitation in insects (Salgado *et al*. 1998), because of its action on nicotinic acetylcholine receptors (nAChRs) (Salgado and Saar 2004).

**Fig. 1:**
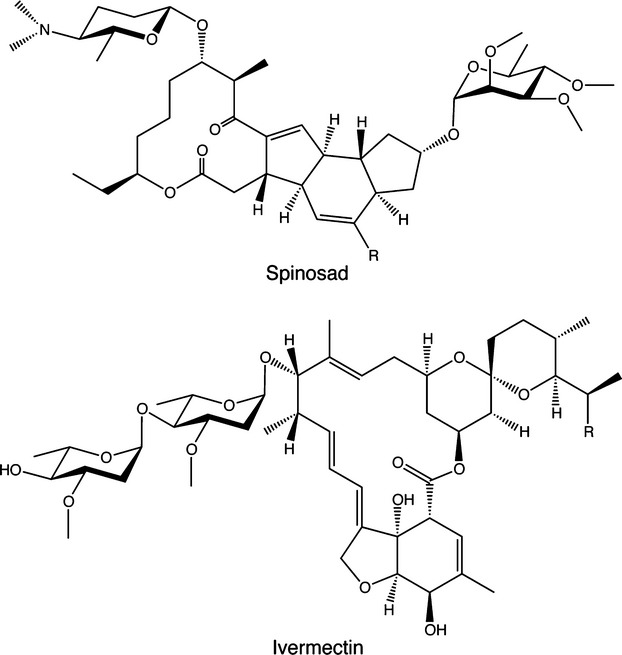
Chemical structure of spinosad and ivermectin. Spinosad is a mixture of spinosin A (in which R = H) and spinosin D (in which R = CH_3_). Also shown is ivermectin, another macrocyclic lactone pesticide. Ivermectin is a mixture of dihydroavermectin B_1a_ (in which R = CH_2_CH_3_) and dihydroavermectin B_1b_ (in which R = CH_3_).

Nicotinic receptors are members of the Cys-loop family of ligand-gated ion channels (Lester *et al*. 2004) and are important neurotransmitter receptor subtypes in both vertebrate and invertebrate species (Millar and Denholm 2007; Millar and Gotti 2009; Jones and Sattelle 2010). The Cys-loop family includes both excitatory (cation-selective) receptors, such as nAChRs, and also inhibitory (anion-selective) receptors (Lester *et al*. 2004). The inhibitory glutamate-gated chloride channel (GluCl), found in several invertebrate species, has close structural similarity to nAChRs and is the target site for ivermectin (Fig. 1), another macrocyclic lactone pesticide (Wolstenholme 2010).

In common with all Cys-loop receptors, nAChRs and GluCls are transmembrane proteins in which five subunits are arranged around a central ion channel pore. Each of the five subunits contains four α-helical transmembrane domains (TM1–TM4), with the second of these domains lining the ion channel pore. The conventional orthosteric agonist binding site is located within the extracellular domain of Cys-loop receptors at the interface between two adjacent subunits (Sine, 2002 #1539). However, several allosteric modulatory sites have also been identified in Cys-loop receptors. In the case of ivermectin, there is clear evidence that it interacts with an allosteric site in the transmembrane domain of GluCls (Hibbs and Gouaux 2011). In addition, ivermectin is an allosteric modulator of nAChRs, and there is evidence that it interacts with nAChRs via the receptor transmembrane region (Krause *et al*. 1998; Collins and Millar 2010). The binding site of spinosad on nAChRs is less well defined, but there is evidence that it also acts an allosteric ligand (Salgado and Saar 2004) at a site that is distinct from the conventional extracellular agonist binding site (Orr *et al*. 2009).

In common with most other pesticides, resistance to macrocyclic lactones such as spinosad and ivermectin is an established problem and one that is increasing as a result of intensive pesticide use (Wolstenholme and Kaplan 2012). Resistance to spinosad has been reported in several insect species (Wolstenholme and Kaplan 2012). For example, there have been reports of resistance in Colorado potato beetle *Leptinotarsa decemlineata* (Mota-Sanchez *et al*. 2006), house fly *Musca domestica* (Shono and Scott 2003) and tobacco budworm, *Heliothis virescens* (Young *et al*. 2003). In such species, there is evidence of resistance being a result of either enhanced metabolism (Markussen and Kristensen 2011) or a consequence of changes in the target site (Roe *et al*. 2010). Studies conducted with the model insect species *Drosophila melanogaster* have implicated the nAChR Dα6 subunit in determining target-site resistance to spinosad (Perry *et al*. 2007; Watson *et al*. 2010). For example, a Dα6 knockout strain of *D. melanogaster* has been shown to confer high levels of resistance to spinosad (Perry *et al*. 2007). In addition, a variety of chemically-induced mutations within Dα6 (generating either truncated proteins or mis-sense mutations) have been found to confer resistance to spinosad (Watson *et al*. 2010). Further evidence indicating that resistance to spinosad can arise through changes to its target-site (the nAChR α6 subunit) is provided by studies with the diamondback moth, *Plutella xylostella* (Baxter *et al*. 2010; Rinkevich *et al*. 2010). Resistance to spinosad in *P. xylostella* has been linked to mis-spliced transcripts of the nAChR α6 subunit resulting in expression of a truncated subunit protein (Baxter *et al*. 2010) and to point mutations generating premature stop codons (Rinkevich *et al*. 2010).

High levels of resistance to the insecticide spinosad have been reported in western flower thrips (*Frankliniella occidentalis*), particularly in areas such as southern Spain, where spinosad has been used intensively to protect greenhouse crops (Bielza *et al*. 2007a, b; Bielza 2008). In this study, we describe work conducted with a previously reported laboratory-selected strain of *F. occidentalis* (R1S) displaying high levels of resistance (resistance ratio > 350 000) to spinosad (Bielza *et al*. 2007b). R1S was selected from a field population of *F. occidentalis* collected in 2003 (in Almeria, Spain), from greenhouses that had been subjected to intensive treatment with spinosad (Bielza *et al*. 2007a, b). Resistance to spinosad in strain R1S has been reported to be autosomal, almost completely recessive and controlled by a single locus (Bielza *et al*. 2007b).

Initial studies of spinosad-resistant *F. occidentalis* indicated that resistance might be associated with target-site changes, rather than enhanced metabolism (Bielza *et al*. 2007a). These findings have prompted us to employ molecular biological techniques to examine the nAChR α6 subunit in *F. occidentalis*. A nicotinic acetylcholine receptor point mutation (G275E), located in the transmembrane region of the receptor has been identified in spinosad-resistant *F. occidentalis*. In addition to its identification in a laboratory-selected strain (R1S), we have also identified this resistance-associated mutation in a recently isolated field population of *F. occidentalis* (Guillén and Bielza 2012). As well as providing evidence for target-site resistance to spinosad in *F. occidentalis*, work described in this article also provides support for the proposal that spinosad acts as a nAChR allosteric modulator via a transmembrane binding site.

## Materials and methods

### Insects

The susceptible strain of *F. occidentalis* (MLFOM) was collected from an organic peach crop from the Murcia region of Spain in 2001 and was maintained subsequently in the laboratory without exposure to insecticide (Bielza *et al*. 2007b). Another population of *F. occidentalis* was collected in 2003 (in Almeria, Spain), from greenhouses that had been subjected to intensive treatment with spinosad, and a resistant strain (R1S) was isolated from this field population after several years of laboratory selection with spinosad (Bielza *et al*. 2007b). A further field population of *F. occidentalis* (MOJO) was collected in 2011 in Almeria, Spain (Guillén and Bielza 2012). Work with *F. occidentalis* was conducted in accordance with procedures reviewed by the Spanish Ministry of Science and Technology.

### Plasmids

The following plasmid expression constructs used in this study have been described previously: human nAChR α7 subunit cDNA in plasmid expression vector pSP64GL (Broadbent *et al*. 2006), mouse 5-HT3A subunit in plasmid pcRK5 (Harkness and Millar 2001) and a subunit chimera containing the extracellular domain of the human α7 subunit fused to the transmembrane domain of the mouse 5-HT3A subunit in plasmid pcDNA3 (Craig *et al*. 2004).

### Molecular cloning of Foα6

Messenger RNA was isolated from approximately 100 spinosad-susceptible *F. occidentalis* (strain MLFOM) using a QuickPrep Micro mRNA purification kit (GE Healthcare, Little Chalfont, UK). Hybrid mRNA/cDNA was synthesized using a First-Strand cDNA Synthesis kit with *Not*I-d(T)_18_ primers (GE Healthcare). Degenerate oligonucleotide primers were designed to two conserved regions of nAChR α6 subunits from other insect species (encoding amino acids DVDEKNQ and WTYDGNQ) and used to amplify a cDNA fragment of 341 bp. The cDNA fragment was ligated into the TA cloning vector pCRII and used to transform *E. coli* One Shot INVαF' competent cells (Invitrogen Life Technologies, Paisley, UK). Individual colonies were grown overnight in LB broth containing ampicillin (50 μg/mL). Plasmid DNA was isolated using GeneJet plasmid miniprep kit (Thermo Fisher Scientific, Waltham, MA, USA) and examined by nucleotide sequencing. Specific oligonucleotide primers were designed to the Foα6 nucleotide sequence and used to isolate longer cDNA fragments by means of 3′ and 5′ rapid amplification of cDNA ends (RACE) using GeneRacer™ kit (Invitrogen Life Technologies). Specific primers were then used to amplify and sequence regions from both susceptible (MLFOM) and resistant (R1S) *F. occidentalis*. This was done with pools of approximately 100 insects (as described above), and also with individual insects. To amplify cDNA from individual insects total RNA was isolated using TRIzol reagent (Invitrogen Life Technologies) using quantities half of that recommended in the manufacturer's protocol. First-strand cDNA was synthesized, using all of the RNA sample extracted from an individual insect, with SuperScriptIII reverse transcriptase (Invitrogen Life Technologies), primed with oligo(dT). Routine PCR amplifications used DreamTaq™ Green PCR Master Mix (Fermentas) and direct nucleotide sequenced performed with specific primers. EMBL nucleotide sequence database accession number: HE965755.

To identify the intron/exon boundaries adjacent to alternative exons 8a and 8b, PCR amplification was performed on genomic DNA isolated from pooled insect samples using TRIzol reagent (Invitrogen Life Technologies) following the manufacturer's protocol. Gene-specific oligonucleotide primers were designed to exon sequences and PCR amplification performed using Long PCR Enzyme Mix (Fermentas, Life Sciences). Amplified DNA fragments were examined either by direct nucleotide sequencing using gene-specific primers or cloned into pCR2.1 vector (Invitrogen Life Technologies) and sequenced with M13 Forward and Reverse primers. All nucleotide sequencing was performed using the Big Dye Terminator Cycle Sequencing kit and ABI Prism 3100-Avant automated sequencer according to the manufacturer's instructions (Applied Biosystems, Life Technologies, Paisley, UK).

### Site-directed mutagenesis and cRNA synthesis

Site-directed mutagenesis was performed with the QuikChange mutagenesis kit (Stratagene, Agilent Technologies, Waldbronn, Germany) with the human nAChR α7 subunit cDNA in pSP64GL (Broadbent *et al*. 2006). Alanine at position 272 (numbering according to Peng *et al*. 1994) was mutated to glutamic acid (A272E) to create a mutation at a position analogous to the G275E mutation in Foα6. Mutated cDNA constructs were verified by nucleotide sequencing, as described above. A full-length *F. occidentalis* nAChR α6 cDNA was amplified from the susceptible (MLFOM) strain using KAPA2G™ Robust HotStart polymerase (KAPA Biosystems, Woburn, MA, USA) and subcloned into pGEMHE. *In vitro* transcription of cRNA, from plasmids encoding Foα6 and human α7, was carried out using mMESSAGE mMACHINE SP6 and T7 transcription kits (Ambion, Life Technologies, Paisley, UK). SP6 and T7 transcription kits were used for pSP6GL-hα7 and pGEMHE-Foα6 respectively.

### Two-electrode voltage-clamp recording

*Xenopus laevis* oocytes were isolated and defolliculated as described previously (Young *et al*. 2007) by treatment with collagenase (2 mg/mL; Worthington, Lakewood, NJ, USA) in calcium-free Barth's solution. Oocytes were injected with 12–25 ng cRNA in a volume of 50 nL into the cytoplasm (Foα7 and hα7 cRNA) or with 0.3 ng cDNA in 18 nL into the oocyte nucleus (pcDNA3-hα7/m5HT3 and pRK5-m5HT3A) using a Drummond variable volume microinjector. After injection, oocytes were incubated at 18°C in Barth's solution containing 0.77 mM CaCl_2_ and supplemented with antibiotics (100 units/mL penicillin, 100 μg/mL streptomycin, 4 μg/mL kanamycin and 50 μg/mL tetracycline). Experiments were performed on oocytes after 3–5 days of incubation. Oocytes were placed in a recording chamber and continuously perfused with a saline solution (115 mM NaCl, 2.5 mM KCl, 1.8 mM CaCl_2_, 10 mM Hepes, pH 7.3). Two-electrode voltage-clamp recordings were performed (with the oocyte membrane potential held at −60 mV), as described previously (Young *et al*. 2007) using a OC-725C amplifier (Warner Instruments, Hamden, CT, USA), PowerLab 8SP and Chart 5 software (AD Instruments, Oxford, UK). Drugs were applied to oocytes using a BPS-8 solenoid valve solution exchange system (ALA Scientific Instruments, Farmingdale, NY, USA). Difficulties were encountered in preparing aqueous solutions of spinosad and in obtaining consistent effects on recombinant nAChRs. Reproducible effects were, however, obtained by preparing, on the day of use, stock solutions (10 mM) of spinosad (Sigma, Poole, UK) in dimethylsulfoxide by sonication for 15 min at 30–40°C. Spinosad was then diluted to its final concentration in saline solution. Consistent effects were observed on α7 nAChRs by pre-incubation with spinosad for 5 min followed by co-application of spinosad with acetylcholine.

### Transient expression of nAChRs in mammalian cells

Human kidney tsA201 cells were cultured in Dulbecco's modified Eagle's medium (Invitrogen Life Technologies) containing 10% foetal calf serum (Sigma), penicillin (100 U/mL) and streptomycin (100 μg/mL) (Invitrogen Life Technologies). Cells were maintained in a humidified incubator containing 5% CO_2_ at 37°C. Cells were transfected using the Effectene reagent (Qiagen, Crawley, UK) according to the manufacturer's instructions. After overnight incubation in Effectene, cells were incubated at 37°C for 24–48 h before being assayed for radioligand binding.

### Radioligand binding

[^3^H]-α-bungarotoxin (56 Ci/mmol; Tocris Bioscience, Bristol, UK) was a gift from Syngenta (Bracknell, UK). Radioligand binding to transiently transfected tsA201 cells was performed essentially as described previously (Lansdell and Millar 2004). Transfected cells were re-suspended in Hank's buffered saline solution (Gibco, Paisley, UK) containing 1% bovine serum albumin and incubated with [^3^H]-α-bungarotoxin for 2 h at 22°C in a total volume of 300 μL. Non-specific binding was determined in the presence of nicotine (1 mM) and carbamylcholine (1 mM). Competition binding experiments were performed by incubating triplicate samples of transfected cells with [^3^H]-α-bungarotoxin (typically, 1 nM), together with a range of concentrations of either spinosad or methyllycaconitine (MLA). Radioligand binding was assayed by filtration onto Whatman GF/A filters (pre-soaked in 0.5% polyethylenimine), followed by rapid washing with phosphate-buffered saline (Oxoid, Basingstoke, UK) using a Brandel cell harvester. Bound radioligand was quantified by scintillation counting. Curves for equilibrium binding were fitted using GraphPad Prism (GraphPad Software, San Diego, CA, USA).

### Statistical analysis

Pair-wise comparisons of statistical significance were performed by Student's unpaired *t*-tests.

## Results

### Cloning of the nAChR Foα6 subunit

**Fig. 2:**
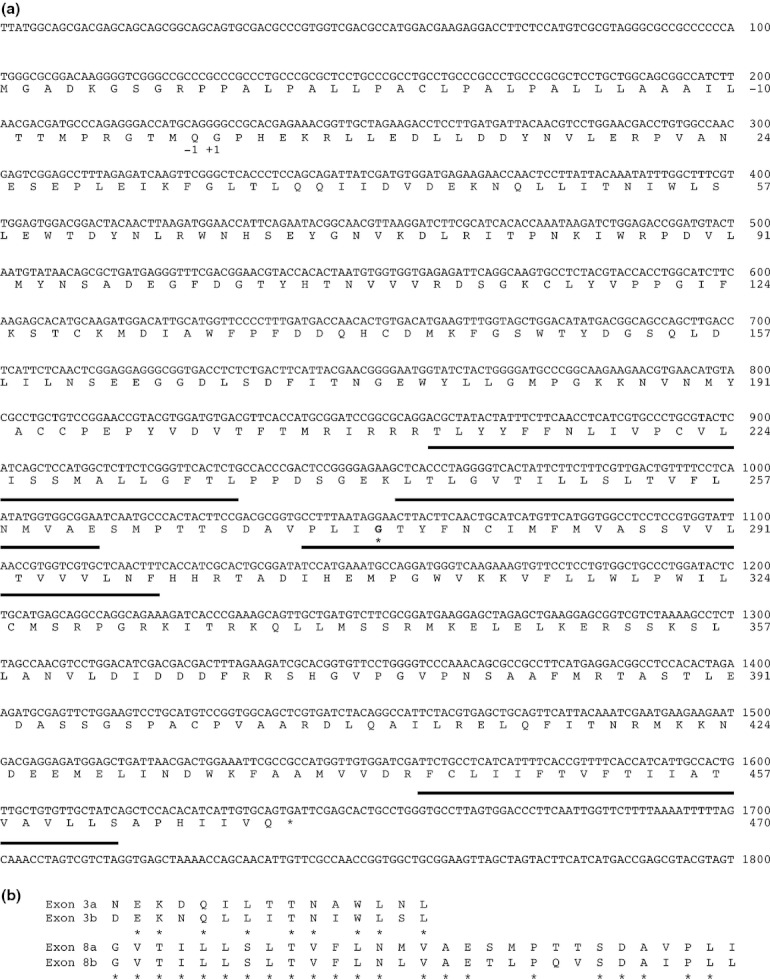
Nucleotide sequence and predicted amino acid sequence of *F. occidentalis* nicotinic acetylcholine receptor (nAChR) Foα6 (Nucleotide sequence database accession number HE965755). (a) The predicted transmembrane domains (TM1–TM4) are underlined. Amino acids are numbered based on the predicted signal sequence cleavage site and the position of the resistance-associated mutation (G275E) is indicated by an asterisk. (b) Amino acid sequence alignment of two alternative exons, 3a/3b and 8a/8b.

**Fig. 3:**
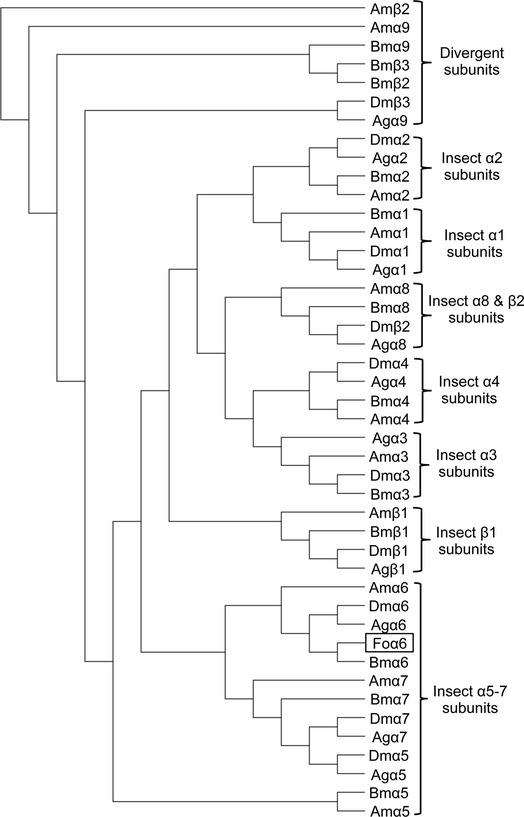
Phylogenetic relationship of insect nicotinic acetylcholine receptor (nAChR) subunits based on predicted amino acid sequence data. The phylogenetic tree was generated in MacVector 12.5.1 using the Best Tree mode and the neighbour-joining analysis method on a ClustalW alignment of selected insect nAChR protein sequences. Species abbreviations are Am: *Apis melifera*, Bm: *Bombyx mori*, Dm: *Drosophila melanogaster*, Ag: *Anopheles gambiae* and Fo: *Frankliniella occidentalis* each with their respective nAChR subtype. Foα6 subunit has closest sequence similarity to other insect nAChR α6 subunits from the nAChR α5-α7 group.

The complete coding sequence of the nAChR α6 subunit from Foα6 was isolated from the spinosad-susceptible strain MLFOM. As described in the Methods, this was achieved by the use of degenerate oligonucleotide primers to isolate partial cDNA fragments, followed by 3′ and 5′ RACE. The nucleotide sequence revealed an open reading frame of 513 amino acids with features typical of a nAChR subunit (Fig. 2) and most closely resembling other insect nAChR α6 subunits (Fig. 3). As has been reported previously for the nAChR α6 subunit cloned from other insect species (Grauso *et al*. 2002; Rinkevich and Scott 2009), we have identified two sets of alternative exons (3a/3b and 8a/8b) in Foα6 transcripts. The transcript we isolated as a full-length cDNA contained alternative exons 3b and 8a (Fig. 2). This corresponds to what has been described as isoform II in *Drosophila melanogaster* (Grauso *et al*. 2002) and *Tribolium casteneum* (Rinkevich and Scott 2009). As is illustrated in Fig. 2, exons 3a and 3b each encode 15 amino acids, of which five amino acids differ in the two alternative exons. Exons 8a and 8b each encode 29 amino acids and differ by seven amino acids (Fig. 2b).

Further cDNA clones were isolated from pools of approximately 100 insects or from individual insects from susceptible (MLFOM) and spinosad resistant (R1S) strains. Transcripts were identified in both susceptible and resistant populations that contained alternative exons 3a/3b and 8a/8b with no evidence to indicate that the frequency of alternative splicing was associated with resistance. However, a glycine codon (GGA; nucleotide position 1051–4 in Fig. 2) was found in all transcripts isolated from susceptible insects, whereas glutamic acid codon (GAA) was found in all transcripts from resistant insects.

Our cDNA cloning of *F. occidentalis* and previously published genomic sequence data from other insect species (Jones and Sattelle 2007; Baxter *et al*. 2010) indicated that this mutation is located at the boundary of exon 9 and the alternatively spliced exons 8a/8b. To confirm the precise location of the mutation, a series of PCR amplifications were performed. The codon for the mutated amino acid was found to span exon 9 and exons 8a/8b, with the resistance-associated mutation (G-A) being at the start of exon 9. In addition, several polymorphisms were identified in susceptible and resistant individuals, but none of these were associated with a particular phenotype.

### G275 is located at conserved position in TM3

Amino acid sequence alignments of Cys-loop ligand-gated ion channel subunits (Table 1) indicated that G275 is located at a conserved position towards the top of the third transmembrane domain (TM3). As explained in the Discussion section, the mutation is predicted to lie four amino acids from the top (the extracellular side) of the α-helical region of the TM3 transmembrane domain. This position is highly conserved, although there are consistent differences between excitatory and inhibitory members of the superfamily. All excitatory (cation-selective) Cys-loop receptors that we have examined contain a glycine (G), alanine (A), isoleucine (I) or serine (S) at this position (Table 1). In contrast, all inhibitory (anion-selective) Cys-loop receptors that we have examined contain an aspartic acid (D). Although the sequences presented in Table 1 are primarily from *Drosophila* and human receptor subunits, we are unaware of any nAChR subunit from any species that contains glutamic acid (E) at the position identified in spinosad resistant *F. occidentalis*. Consequently, the occurrence of a glutamic acid at this position in the spinosad-resistant Foα6 subunit (an excitatory receptor subunit) is extremely unusual and is consistent with it being a resistance-associated mutation.

**Table 1:** Amino acid sequence surrounding the TM3 4′ position (bold) in cation-selective (upper panel) and anion-selective (bottom panel) Cys-loop receptors. Based on previously published alignments of Cys-loop receptors (Lester *et al*. 2004; Hibbs and Gouaux 2011)

*F. ocidentalis* α6	DAIPLL**G**TYFNCI
*D. melanogaster* α1	LTVPLL**G**KYLLFT
*D. melanogaster* α2	LALPLL**G**KYLLFT
*D. melanogaster* α3	LVVPLL**G**KFVLFT
*D. melanogaster* α4	LVVPLL**G**KYLIFA
*D. melanogaster* α5	DAVPLL**G**TYFNCI
*D. melanogaster* α6	DAVPLI**G**VTILLS
*D. melanogaster* α7	DAVPLL**G**KYFNCI
*D. melanogaster* β1	LVLPLI**A**KYLLFT
*D. melanogaster* β2	LAVPLL**G**KYLLFT
Human nAChR α1	SAVPLI**G**KYMLFT
Human nAChR α2	LVIPLI**G**EYLLFT
Human nAChR α3	LVIPLI**G**EYLLFT
Human nAChR α4	LVIPLI**G**EYLLFT
Human nAChR α5	KVIPLI**G**EYLVFT
Human nAChR α6	LVVPLV**G**EYLLFT
Human nAChR α7	DSVPLI**A**QYFAST
Human nAChR α9	ENVPLI**G**KYYIAT
Human nAChR α10	ESVPLI**G**KYYMAT
Human nAChR β1	LSVPII**I**KYLMFT
Human nAChR β2	LDVPLV**G**KYLMFT
Human nAChR β3	KVIPLI**G**EYLLFI
Human nAChR β4	LDVPLI**G**KYLMFT
Human nAChR γ	QAVPLI**S**KYLTFL
Human nAChR δ	MAIPLI**G**KFLLFG
Human nAChR ε	LSVPLL**G**RFLIFV
Human 5-HT3A	IGTPLI**G**VYFVVC
Human 5-HT3B	GSTPLI**G**HFFTIC
	
*C. elegans GluCl*	SYIKAI**D**VWIGAC
*D. melanogaster RDL*	SYVKSI**D**VYLGTC
*D. melanogaster GRD*	SYPTAL**D**FFVFLS
*D. melanogaster LCCH3*	SYVKAI**D**IYLVMC
*D. melanogaster HisCl*	SYLKAV**D**AFMSVC
*D. melanogaster GluCl*	SYTKAI**D**VWTGVC
Human GABA α1	AYATAM**D**WFIAVC
Human GABA α2	AYATAM**D**WFIAVC
Human GABA α3	AYATAM**D**WFIAVC
Human GABA α4	SYLTAM**D**WFIAVC
Human GABA α5	AYATAM**D**WFIAVC
Human GABA α6	SYATAM**D**WFIAVC
Human GABA β1	PYVKAI**D**IYLMGC
Human GABA β2	PYVKAI**D**MYLMGC
Human GABA β3	PYVKAI**D**MYLMGC
Human GABA δ	SAIKAL**D**VYFWIC
Human GABA ρ1	SYIKAV**D**IYLWVS
Human GABA ρ2	SYVKAV**D**IYLWVS
Human GylR α1	SYVKAI**D**IWMAVC
Human GylR α2	SYVKAI**D**IWMAVC
Human GylR α3	SYVKAI**D**IWMAVC
Human GylR β	SYVKAL**D**VWLIAC

### Comparison to the ivermectin-binding site in GluCl

**Fig. 4:**
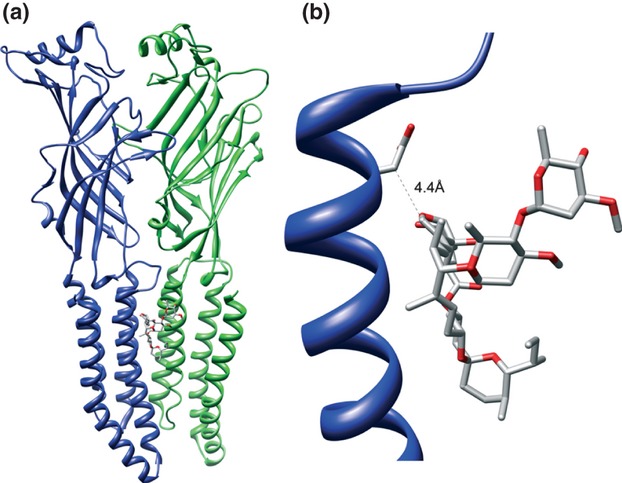
The location of bound ivermectin in the *C. elegans* glutamate-gated chloride channel (GluCl) crystal structure. (a) For clarity, only two of the five subunits are illustrated (in blue and green). Also shown is ivermectin, bound to the GluCl transmembrane domain. (b) The upper portion of the M3 transmembrane helix of GluCl is shown, together with the position of bound ivermectin. Also illustrated is the side chain of the amino acid (aspartic acid; D) located at a position analogous to the resistance-associated mutation (G275E) in Foα6. The side chain of the aspartic acid residue in GluCl is within 4.4 Å of the bound ivermectin.

Although the site at which spinosad interacts with nAChRs remains unknown with certainty, it seems plausible that it might interact in a manner similar to that by which other macrocyclic lactones interact with Cys-loop neurotransmitter-gated ion channels. A high resolution X-ray structure has been determined recently for the glutamate-gated chloride channel (GluCl) from *Caenorhabditis elegan*s, co-crystallized with ivermectin (Hibbs and Gouaux 2011). Ivermectin, like spinosad, is a macrocyclic lactone (Fig. 1) that is widely used as a pesticide (Raymond and Sattelle 2002; Wolstenholme 2010). From the GluCl structure, it is apparent that ivermectin interacts at a binding site located at the periphery of the transmembrane region towards the extracellular side of the lipid bilayer (Hibbs and Gouaux 2011) (Fig. 4). It makes close contact (by hydrogen-bonding and van der Waals interactions) with the TM1, TM2 and TM3 (Hibbs and Gouaux 2011). As a result of the sequence similarity between nAChRs and GluCls (Hibbs and Gouaux 2011), it is possible to identify the position in the GluCl structure that is analogous to the resistance-associated mutation in the nAChR Foα6 subunit. As is illustrated in Fig. 4, the analogous amino acid in the *C. elegans* GluCl is located at the top of the TM3 transmembrane helix and is in close proximity to the bound ivermectin. The aspartic acid side chain of GluCl is within 4.4 Å of the tetrahydrofuran ring of ivermectin. It is also one of small number of amino acids in GluCl that have been identified as making van der Waals interactions with ivermectin (Hibbs and Gouaux 2011).

### Functional characterization of TM3 mutation in α7 nAChRs

The full-length Foα6 cDNA was expressed in *Xenopus* oocytes, but did not generate functional nAChRs, either when expressed alone or with other nAChR subunits. In addition, co-expression of Foα6 with the nAChR chaperone RIC-3 (Lansdell *et al*. 2008; Millar 2008) failed to facilitate functional expression. To some extent, this was not unexpected, given the widely acknowledged difficulties in expressing insect nAChRs in heterologous expression systems (Millar 1999; Millar and Lansdell 2010). However, we were able to detect functional homomeric nAChRs routinely, when the closely related human α7 subunit was expressed in oocytes, as has been described previously (Couturier *et al*. 1990). Consequently, the effect of the analogous mutation (A272E) was examined in the human nAChR α7 subunit (the human α7 subunit contains an alanine, rather than a glycine at this position; Fig. 2).

**Fig. 5:**
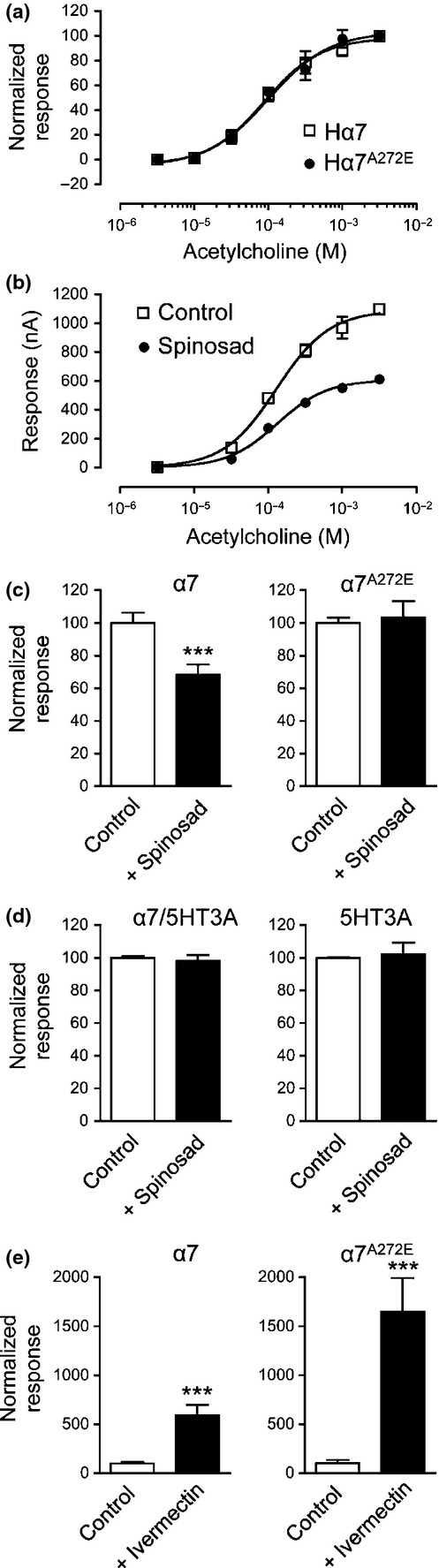
Functional characterization of wild-type and mutated human α7 nicotinic acetylcholine receptors (nAChRs) expressed in *Xenopus* oocytes. (a) Acetylcholine dose-response curves for wild-type and mutant (A272E) human nAChR α7 subunit. Data points are means (± SEM) of three separate determinations. (b) Dose-response data from a single oocyte (but representative of three independent experiments from different oocytes) in which the effect of spinosad was examined on responses to a range acetylcholine concentrations. Data points are means of two to three responses from a single oocyte. (c) Bar graphs indicating the effect of spinosad on acetylcholine-induced responses on wild-type α7 nAChRs (*n* = 21) and mutated (A272E) α7 nAChRs (*n* = 16). (d) Bar graphs indicating the effect of spinosad on agonist (acetylcholine or 5-hydroxytryptamine)-induced responses on α7/5HT3A subunit chimera (*n* = 6) and the 5-HT_3A_ receptor (*n* = 10). (e) Bar graphs indicating the effect of ivermectin on acetylcholine-induced responses on wild-type α7 nAChRs (*n* = 11) and mutated (A272E) α7 nAChRs (*n* = 10). In all experiments (c–e), modulators (spinosad or ivermectin; 30 μM) were pre-applied for 5 min and then co-applied with agonist. Data (c–e) obtained with spinosad or ivermectin (filled bars) are normalized to responses to acetylcholine (100 μM) or 5-hydroxytryptamine (100 μM) in the absence of modulator (open bars) and are means ± SEM from 6 to 21 independent paired experiments. ***p < 0.001.

The A272E mutation was introduced into the human α7 subunit by site-directed mutagenesis and the mutated cDNA expressed in *Xenopus* oocytes and examined by two-electrode voltage-clamp recording. Acetylcholine dose-response curves were determined for both wild-type receptors and α7 containing the A272E mutation (Fig. 5a). The A272E mutation had no significant effect on either *EC*_50_ values for acetylcholine (104 ± 21 μM for wild-type and 134 ± 47 μM for the mutant; *n* = 3) or on Hill coefficient (1.1 ± 0.2 for wild-type and 1.1 ± 0.3 for the mutant; *n* = 3). In contrast, the A272E mutation had a dramatic effect on modulation of α7 nAChRs by spinosad (Fig. 5b). Spinosad was found to be an inhibitor of human α7 nAChRs, causing a substantial reduction in acetylcholine-evoked responses (Fig. 5b). Spinosad (30 μM) had no significant effect on the *EC*_50_ value for acetylcholine, but caused a reduction in the maximum acetylcholine response (Fig. 5b). Responses to a maximal concentration of acetylcholine (100 μM) were significantly reduced in the presence of spinosad (68.1 ± 6.4% compared with responses in the absence of spinosad; *n* = 21; *p* < 0.001; Fig. 5c). In contrast, spinosad had no significant effect on α7 nAChRs containing the A272E mutation (Fig. 5c).

Whereas, spinosad is an antagonist of α7 nAChRs, ivermectin (another macrocyclic lactone; Fig. 1) has been shown previously to be a positive allosteric modulator of this receptor (Krause *et al*. 1998; Collins and Millar 2010). We therefore examined whether the α7 nAChR A272E mutation also had an effect on modulation by ivermectin of acetylcholine responses. On wild-type α7 nAChRs, ivermectin (30 μM) potentiated responses to acetylcholine (100 μM) by 5.9 ± 1.0 fold (*n* = 11), but it caused a significantly larger potentiation (16.5 ± 3.6 fold; *n* = 10; *p* < 0.005) of α7 nAChRs containing the A272E mutation (Fig. 5e).

In contrast to the effects of spinosad on wild-type α7 nAChRs, spinosad had no significant effect on the amplitude of agonist responses with 5-HT_3A_ receptors or with a previously described subunit chimera (Craig *et al*. 2004) that contains the extracellular domain of the α7 subunit fused to the transmembrane domain of the 5-HT3A subunit (Fig. 5d). These findings are consistent with the hypothesis that spinosad is an allosteric antagonist of human α7 nAChRs and interacts non-competitively at a site other than the conventional extracellular orthosteric binding site, perhaps similar to the transmembrane binding site of ivermectin (Collins and Millar 2010; Hibbs and Gouaux 2011).

### Competition radioligand binding

**Fig. 6:**
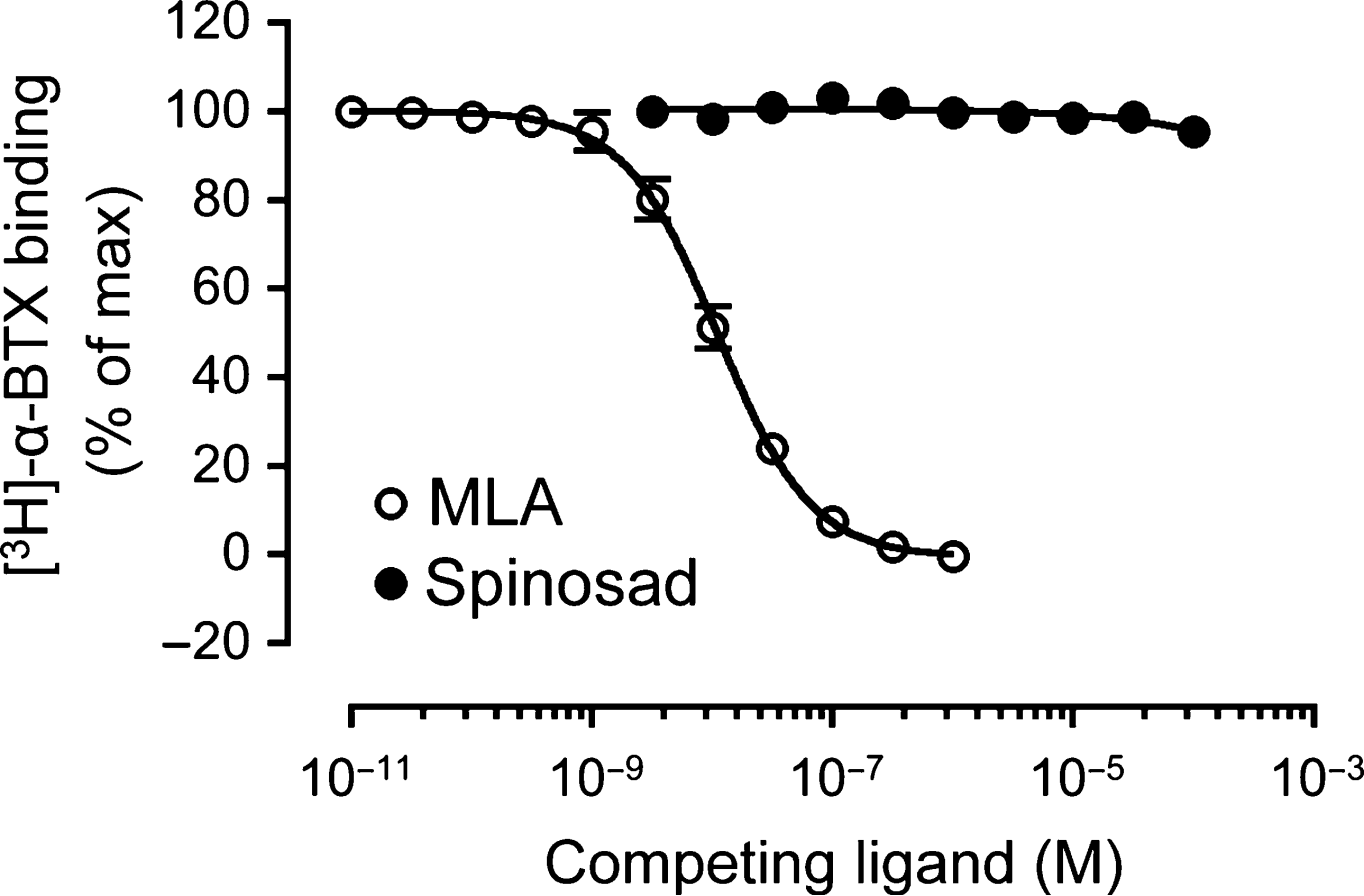
Competition radioligand binding on tsA201 cells transiently expressing with human α7 nicotinic acetylcholine receptors (nAChRs). Equilibrium radioligand binding was performed with [^3^H]-α-bungarotoxin (1 nM). Spinosad caused no significant displacement of [^3^H]-α-bungarotoxin binding, whereas MLA caused complete displacement of specific radioligand binding (*K*_i_ = 4.2 ± 0.2 nM). Data points are means of triplicate samples (± SEM) from a single experiment, and data are typical of three independent experiments.

To test the hypothesis that spinosad is a non-competitive (allosteric) antagonist of human α7 nAChRs, we examined whether it is able to displace the binding of [^3^H]-α-bungarotoxin to the receptor. No significant displacement of [^3^H]-α-bungarotoxin binding was observed, even at the maximum concentration of spinosad tested (100 μM; Fig. 6). This suggests that spinosad does not bind competitively at the orthosteric nicotinic ligand-binding site, a result that is consistent with our functional data. In contrast to spinosad, the competitive antagonist MLA caused complete displacement [^3^H]-α-bungarotoxin from α7 nAChRs (Fig. 6). The calculated *K*_i_ value for MLA was 4.2 ± 0.2 nM (*n* = 3), similar to previous estimates of the affinity of MLA for α7 nAChRs (Davies *et al*. 1999).

### Identification of the G275E mutation in a field population of *F. occidentalis*

As described, we have identified a resistance-associated mutation (G275E) in a laboratory-selected strain of *F. occidentalis* (R1S). As has been described previously, R1S was selected from a field population (R1) that, itself, had high levels of resistance to spinosad (Bielza *et al*. 2007a). This would suggest that the G275E mutation may have been present in the original population (collected in 2003). However, to examine whether this mutation is present in field populations, RT-PCR was performed on individual *F. occidentalis* from a field population (MOJO) collected in 2011 from Almeria, Spain (Guillén and Bielza 2012). This is the same location that the original resistant population was collected and a region in which severe problems of resistance to spinosad have been reported. Initial studies have revealed that this field population (MOJO) is a mixture of homozygous wild-type (glycine at position 275), mutant (G275E) and heterozygous individuals. Further work will be required to determine the frequency of this mutation within this population and also the geographical spread of the mutation. Nevertheless, this provides clear evidence that the G275E mutation is present within field populations of *F. occidentalis* in southern Spain.

## Discussion

There is extensive evidence to indicate that insecticide resistance can arise as a consequence of enhanced metabolic detoxification (Scott 1999). However, in recent years, there has been increasing evidence that resistance can also occur as a consequence of mutations in the insecticide target site. The phenomenon of target-site resistance is well established for several insecticides, including organophosphates, acting on acetylcholine esterase and pyrethroids, acting on voltage-gated sodium channels. In contrast, it is only relatively recently that target-site resistance has been reported for insecticides acting on nAChRs (Millar and Denholm 2007; Wolstenholme and Kaplan 2012). For example, point mutations altering single amino acids in nAChR α or β subunits have been described that are associated with resistance to neonicotinoid insecticides (Liu *et al*. 2005; Bass *et al*. 2011).

Evidence has also accumulated in recent years to indicate that resistance to spinosad can occur as a result of changes in its target site, the nAChR (Wolstenholme and Kaplan 2012). Disruption of the Dα6 subunit in *Drosophila* has been reported to confer resistance to spinosad (Perry *et al*. 2007). In addition, mis-spliced α6 transcripts and truncated α6 subunits in *Plutella xyostella* are associated with resistance to spinosad (Baxter *et al*. 2010; Rinkevich *et al*. 2010). There is also evidence for resistance to spinosad in *Drosophila* because of chemically-induced mutations resulting in truncated or non-functional Dα6 subunits (Watson *et al*. 2010). In contrast, this study has identified a resistance-associated mutation located at a position close to a plausible binding site for spinosad. In addition, when this mutation is introduced into a closely related vertebrate nAChR, it generated a functional receptor with reduced sensitivity to spinosad, but no apparent effect on the potency of the endogenous agonist acetylcholine. In this respect, the spinosad resistance-associated mutation identified in Foα6 resembles a previously characterized nAChR mutation that is associated with resistance to neonicotinoid insecticides (Liu *et al*. 2005), which has a profound effect on agonist activation by neonicotinoids but only minimal effects on agonist activation by acetylcholine (Liu *et al*. 2006).

The best structural data available for the transmembrane region of a native nAChR are that generated by electron diffraction studies conducted with receptors purified from *Torpedo* electric organ (Unwin 2005). However, on the basis of higher-resolution X-ray diffraction data from bacterial ligand-gated ion channels and GluCl, it has been proposed that the assignment of amino acids in the TM3 domain of the *Torpedo* nAChR is of register by four residues, equivalent to about one turn of the α helix (Corringer *et al*. 2010; Hibbs and Gouaux 2011). On the basis of this information, we have assigned the position of the amino acid that is mutated in spinosad-resistant *F. occidentalis* as being the fourth amino acid from the top of the TM3 helix.

As is illustrated in Fig. 2, the position of the G275E mutation in Foα6 is at a position analogous to an aspartic acid (D) in GluCl. Not only is this aspartic acid residue in very close proximity (4.4 Å) to bound ivermectin in the GluCl crystal structure, it is also one of the amino acids that is involved in forming a van der Waals interaction with ivermectin (Hibbs and Gouaux 2011). Given the known location of the ivermectin biding site in GluCl, it seems plausible that the G275E mutation might be in close proximity to the spinosad binding site on nAChRs. This is consistent with our data indicating that the A272E mutation in human α7 nAChRs has an effect on the modulation of agonist responses by both spinosad and ivermectin (Fig. 5). The finding that spinosad does not modulate agonist responses in a subunit chimera containing the extracellular domain of the nAChR α7 subunit but the transmembrane domain of the 5-HT3A subunit (Fig. 5d), is also consistent with spinosad binding at a transmembrane location, similar to the known binding site of ivermectin on GluCl (Hibbs and Gouaux 2011). Furthermore, competition binding data (Fig. 6) provide further support for the conclusion that spinosad binds at a site other than the conventional orthosteric nicotinic binding site and is in agreement with previous evidence indicating that spinosad modulates nAChRs by interacting with a site distinct from the conventional agonist binding site (Orr *et al*. 2009). There are reports that spinosad acts as an agonist on some insect nAChRs (Salgado and Saar 2004; Watson *et al*. 2010). This is entirely consistent with spinosad acting via an allosteric transmembrane site, given the recent evidence indicating that nAChRs can be activated by allosteric agonists binding to a transmembrane site (Gill *et al*. 2011, 2012).

Further evidence that the TM3 domain of Cys-loop receptors is important in the binding of macrocyclic lactones comes from studies conducted with an insect GluCl channel and a vertebrate glycine receptor (GlyR). Both studies have examined mutations influencing ivermectin and both have identified an amino acid in TM3 that is predicted to lie four amino acids below that of the G275E mutation in Foα6. Significantly, as four amino acids corresponds to one turn of an α-helix, the residue identified in this study and that identified in the insect GluCl and vertebrate GlyR are predicted to have side chains pointing in the same approximate orientation. A study investigating resistance to ivermectin identified a resistance-associated point mutation (G323D) in the TM3 domain of a GluCl subunit from the two-spotted spider mite *Tetranychus urticae* (Kwon *et al*. 2010). Interestingly, like the mutation that we have identified in Foα6, the mutation identified in the GluCl also corresponds to a change from a glycine to an acidic residue (Kwon *et al*. 2010). In addition, studies with the vertebrate GlyR have demonstrated that the amino acid in GlyR α1 subunit equivalent to G323 the GluCl in (A228) can confer either enhanced sensitivity (A288G) or reduced sensitivity (A288T) to ivermectin (Lynagh and Lynch 2010; Lynagh *et al*. 2011).

Considerable problems have been encountered in expressing insect nAChRs in heterologous expression systems (Millar 1999; Millar and Lansdell 2010). Indeed, such problems have been reported in connection with α6 subunits cloned from other insect species (Lansdell and Millar 2004). In situations where functional expression has been achieved with α6-containing nAChRs, it has been reported to be inconsistent and often unsuccessful (Watson *et al*. 2010). Attempts were made to express the cloned Foα6 subunit in *Xenopus* oocytes, but these were unsuccessful. Because of the relative ease with which the vertebrate nAChR α7 subunit can be expressed as a functional homomeric receptor, it has been used extensively as a model for investigating mutations affecting neonicotinoid insecticides (Matsuda *et al*. 2000; Shimomura *et al*. 2002, 2003; Amiri *et al*. 2008). By comparing the functional properties of the vertebrate α7 nAChR containing a TM3 A272E mutation, it has been possible to demonstrate that this mutation has no significant effect on acetylcholine potency, as might be expected for a mutation located far from the extracellular binding site for acetylcholine. The absence of an effect on acetylcholine agonist potency is similar to the effects that have been described previously for a target-site mutation associated with resistance to neonicotinoid insecticides (Liu *et al*. 2006). Significantly, the resistance-associated mutation identified in Foα6 has also been found to abolish modulation of human α7 nAChRs by spinosad. Although spinosad appears to act as an agonist of insect nAChRs (Salgado and Saar 2004; Watson *et al*. 2010), with features similar to that of an allosteric agonist (Gill *et al*. 2011), we have found that spinosad is an antagonist of human nAChRs. This difference in the influence of spinosad on two different nAChRs is not unexpected. The chemically related macrocyclic lactone ivermectin is a positive allosteric modulator of human α7 nAChRs (Krause *et al*. 1998), but a single point mutation in the transmembrane region can convert it from a positive to a negative allosteric modulator (Collins and Millar 2010). Consequently, it is plausible that spinosad might interact at a similar transmembrane site in insect and human nAChRs but have opposing effects. What is significant is that a A272E mutation introduced into the human α7 nAChR abolishes the modulatory effects of spinosad, perhaps through a direct action on its binding and, consequently, this mutation might reasonably be expected to have a similar effect on the interaction of spinosad with insect nAChRs. It seems likely that both spinosad and ivermectin modulate Cys-loop receptors by interacting with an allosteric transmembrane site. Furthermore, it appears that both of these macrocyclic lactones, depending on the receptor they are acting upon, can exert a range of allosteric modulatory effects. These include positive allosteric modulation (potentiation), negative allosteric modulation (non-competitive antagonism) and allosteric agonist activation (activation in the absence of a conventional orthosteric agonist). As has been demonstrated recently for allosteric modulators of vertebrate nAChRs, it appears that all of these effects can potentially occur through transmembrane allosteric binding sites (Young *et al*. 2008; Collins *et al*. 2011; Gill *et al*. 2011).

In summary, we have identified a resistance-associated point mutation in the transmembrane domain of the Foα6 subunit, in a position analogous to the known binding site for ivermectin in a related Cys-loop receptor. Studies with the vertebrate nAChR α7 subunit provide evidence to suggest that the TM3 G275E mutation identified in Foα6 may be responsible for conferring target-site resistance to spinosad by exerting a selective effect on modulation by spinosad at its presumed allosteric binding site, together with a negligible effect on acetylcholine acting at its extracellular orthosteric binding site.

## Abbreviations

GluCl: glutamate-gated chloride channel
MLA: methyllycaconitine
nAChR: nicotinic acetylcholine receptor
